# Trust-building vs. “just trust me”: reflexivity and resonance in ethnography

**DOI:** 10.3389/fsoc.2023.1069305

**Published:** 2023-04-24

**Authors:** Allison J. Pugh, Sarah Mosseri

**Affiliations:** ^1^Department of Sociology, University of Virginia, Charlottesville, VA, United States; ^2^Department of Work and Organisational Studies, University of Sydney Business School, Sydney, NSW, Australia

**Keywords:** resonance, reflexivity, ethnographic accountability, culture, trust

## Abstract

Amidst a perceived credibility crisis, recent scholarship has challenged basic norms of how ethnographies are conducted. This article identifies, underlying these critiques, a “trust me” fallacy that misunderstands ethnography as requiring blind trust in the researcher, leading to proposed reforms that promote extractive research practices by treating truths as raw commodities to be traded in for credibility. We argue such practices are unlikely to resolve critics' concerns, and at the same time, they challenge the ethnographic capacity for resonance. Building on recent work in cultural sociology, we elaborate and refine a “textured model of resonance” to capture one of ethnography's unique contributions: excavating ambivalence, plurality and complexity. We conclude by noting how time-honored practices of reflexivity, honed through productive dialogue among practitioners, address issues of trust and reliability without threatening what ethnography does well.

## Introduction

A flurry of scholarship has arisen around contemporary practices of ethnography, suggesting reforms designed to address a perceived credibility crisis. Yet we argue that most of these proposals reflect a core misunderstanding of what is valuable about ethnography, particularly its potential for resonance. This article makes two core contributions: first, we identify a “trust me” fallacy, through which challengers read ethnographers as enjoying undue benefits of trust, a fallacy underlying current critiques of ethnographic research. The “trust me” fallacy misses how credibility is painstakingly layered into historically grounded ethnographic praxis and is also highly visible within the text. Second, after reviewing problems with recent reforms coming out of this misplaced critique, we then explore a vital dimension of ethnographic value: the capacity for resonance. Building on recent work in cultural sociology, we elaborate and refine the concept of resonance in ethnography, proposing a “textured” model. We illustrate how talking about resonance helps clarify some of the more elusive qualities that constitute good ethnographies. We conclude by noting how time-honored practices of reflexivity, honed through productive dialogue among practitioners, do not pose the same threats to resonance as the proposed reforms.

## The “trust me” fallacy

Numerous papers have been published in the past few years, each offering proposals for improving transparency in qualitative research, and ethnography in particular. We see these papers as attempts to address a perceived credibility crisis in ethnography. The starting point for this crisis traces back to an uproar in 2015 over the non-replicability of some survey research (begun in psychology (Open Science Collaboration, 2015) but extending to political science (e.g., Broockman et al., 2015) and other fields [e.g., economics (Camerer et al., 2016)]). The turn to ethnography occurred amid debate over Goffman's (2014) *On the Run*, an account of young Black men in Philadelphia and how over-policing made them a fugitive class. New calls for ethnographic reform arose as a result, some from outside the discipline (Lubet, 2018), but others from practitioners themselves (Pool, 2017; Jerolmack and Murphy, 2019; Murphy et al., 2021).

Implicit within the discourse on reforming ethnographic practice is an anxiety about trust. Hancock et al. (2018) clearly articulate this apprehension while reviewing challenges to validity in ethnographic research, writing, “the reader is (often implicitly) expected to trust the accuracy of the observer and trust that he or she rendered or translated experiences faithfully” (321). While weighing the benefits and risks of identifying participants, Contreras' (2019) reveals how the impact of the discourse, whether intended or not, brings ethnographers' trust into question. Of his ultimate decision to show images of his participants in an academic presentation, a move that compromises their confidentiality, he says, “I refused to be called a charlatan, a cheat, an imposter, or a liar. I wanted to be regarded as a scholar with integrity.” In a recent article published in the *Annual Review of Sociology*, Murphy et al. (2021) suggest that “ethnographic conventions (e.g., deidentifying people and places and shielding or destroying fieldnotes) *arouse suspicion* that the researcher may have something to hide (Kaminer, 2012; Singal, 2015)” (42) and that “ethnographers who insist that standards of replication and verification cannot and should not apply to their work (Tsai et al., 2016) and that ethics prevents them from sharing their data or disclosing the names of the people and places they study, are being *greeted with greater skepticism*” (42, *emphasis added*).

These statements reflect what we call the “trust me” fallacy within calls for reform, or the assumption that readers of ethnographic texts are being asked to blindly trust the individuals that produce them, the inherent risk being that the ethnographer, willfully or not, will somehow dupe the naïve audience. To meet this alleged threat, reformers offer verification solutions. And the stakes are high: Jerolmack and Murphy (2019, p. 819) warn “ethnography, we believe, risks marginalization if it continues to ignore scholarly and public demands for greater transparency”. We contend, however, that reformers have misdiagnosed the issue. Concern regarding individual researchers' trustworthiness belies the more fundamental point of contention: how do we evaluate interpretive work, especially given its focus on truths that are relational, partial, multiple and contradictory?

We identify two blind spots within existing reforms: (1) an undersocialized understanding of facts; and (2) an uncritical approach to transparency. Consequently, these reforms promote what we consider to be, in some cases, ethically dubious practice and risk undermining the value of ethnographic research. We contend it is crucial to confront these erroneous conceptions before they become standard practice, particularly as pressure to conform will likely be inversely felt by those with the least power, posing a disproportional burden for graduate students, junior scholars and those who have been historically marginalized within the academy.^1^

### The problem with facts

“We may call truth [that which] we cannot change,” wrote Arendt (2005, p. 313), arguing that factual truths were those “related to other people…even if [they] occur in privacy” (301), the “other people” recalling Durkheim's notion of “social facts,” which he considered collective forces or entities both external to individuals but also constraining them (e.g., social norms, values and institutions). Thus, facts—or factual truths—are deeply embedded in social contexts. The emphasis on research transparency and replication, however, rests upon an underlying assumption that data are self-evident, able to meaningfully stand apart from the contexts in which they were gathered. This view, of course, contradicts the very strength of ethnographic data, in which realities are multiple, contradictory and contextually bound [see Pratt et al. (2020) for a similar point].

To be sure, reformers—especially experienced ethnographers (e.g., Duneier, 1999; Desmond's, 2016; Jerolmack and Murphy, 2019; Murphy et al., 2021)—wrestle with the important nuances of this point, acknowledging challenges to reproducibility and reducibility in interpretive work. And indeed, methodological debate and technological innovations (e.g., machine learning) have begun to chip away at the hegemony of traditional positivist assumptions and practices across the discipline, with even quantitative researchers starting to openly adopt more idiographic, inductive, and interpretive approaches (Babones, 2016; Nelson, 2020).^2^ Nevertheless, recent ethnographic reform proposals rely on an underlying assumption of data extractability, and we argue, if enacted as standard practice, they pose the risk of overstepping the reformers' more carefully crafted intentions.

To take one popular proposal, fact-checking, in which a third party vouchsafes the accuracy of one's data (Desmond's, 2016), what are the sorts of “facts” that these external arbiters are checking, and to what end? We argue that there are multiple kinds of facts, and that among these, at least three kinds—*contradictory facts, sedimentary facts and motivational facts—*are both essential to the knowledge produced *via* ethnography, and yet not very well suited to a fact-checking paradigm.

The first kind of facts particularly troublesome for fact-checkers are *contradictory facts*, which arise when what people say is contradicted by what they do, or when data collected *via* one method contradicts data collected *via* another. We consider these contradictory facts findings, as opposed to evidence of poor data quality or a failed attempt at corroboration, because what people say and what people do can both be “factual truths”, but also in opposition to each other. For example, in Pugh's (2009) ethnography of parents and children wrestling with consumer culture, she found that low-income parents bought very little for their children, but often claimed they bought a lot, while affluent parents talked about how little they bought for their children, who nonetheless had very well-stocked bedrooms. From these facts, she made these interpretations: she dubbed these twin practices “symbolic indulgence” and “symbolic deprivation,” and she argued that each reflected what the parents were positioning themselves against—the incompetent parent who couldn't provide, or the materialistic parent who couldn't say no. It is a classic move in ethnography, relying on both interviews and fieldwork observations to juxtapose talk and action while trying to make sense of both, treating subjects with what Fine (2019, p. 831) called “skepticism without derision” (see also Jerolmack and Khan, 2014).

Verifying this contradictory fact would be close to impossible, because to do so would be essentially the same as conducting the ethnography, requiring interviewing and observing in the homes without disclosing the contradiction sought, so as not to inspire parents to run around the home hiding toys or to defensively change their claim. But equally important, checking contradictory facts would be quite discourteous, scorning rather than honoring the gift of participation. Recall in Hochschild's (1989) *The Second Shift*, when we heard the click, click, click of Nancy Holt's footsteps going to the laundry after dinner, exposing the inequality behind her egalitarian claim to split the chores with her husband Evan between the “upstairs” and “downstairs.” A fact-checker could perhaps get Nancy to admit that actually, she does most of the chores, and yes, she had said they split it, but there would be some pain in forcing that admission.^3^ These contradictions are often doing something for the informant—they represent a discursive stance that papers over the irreconcilable cultural conflicts they straddle—and a direct confrontation is intrusive, not to mention obnoxious and even threatening, all to meet the researcher's goal rather than the informant's.

The service that contradictory facts are doing for informants—that cultural reconciliation—is, we would argue, sociological gold, one of the most valuable findings ethnographers have to offer (see Vaughan, 1996; Fields, 2008; Van Cleve's, 2016; Clair, 2020 for other examples among many). Contradictory facts demonstrate not just informants' acts but the meaning they make from them; they allow us to see the pressures people feel from the colliding demands of their social world, and how they manage that collision. All in one paradox, we can see both yoke and yearning, and how people bend to one without relinquishing the other, a process rendered the more powerful because of people's reluctance to admit it (Pugh, 2013). There are circumstances under which the researcher might probe such contradictions without undue harm. But in many cases, forcing informants to acknowledge inconsistencies explicitly, in addition to being difficult and insensitive, would threaten the ability to achieve an important signifier of value in qualitative research: namely, the demonstration of what Small and Calarco (2022) describe as “cognitive empathy”—one of their five indicators of good qualitative research (alongside heterogeneity, palpability, follow-up, and self-awareness). Cognitive empathy, they argue, is about understanding participants' beliefs holistically, from origination to their ongoing pragmatic value, and in the case of contradictory facts, they help ethnographers understand the work people do to cope with or resolve everyday tensions produced by a group's structure and culture.

The truth of *sedimentary facts* is more verifiable, but what makes such facts sedimentary is they are individually very small, yet when combined they provide evidence for a particular claim; sedimentary facts also offer the details and texture of lived experience—what Small and Calarco call “palpability”—that lift the narrative off the page, creating, for the reader, a connective virtual space for orienting themselves to or within the story. A source of not only cognitive empathy but also heterogeneous understanding, sedimentary facts are the building blocks of good ethnographies.

The value of sedimentary facts is illustrated in Mosseri's (2019) ethnography investigating the intersections of intimacy, insecurity and inequality in contemporary work. Detailing life inside a busy Manhattan restaurant she dubs The Jones, Mosseri describes how Ken, a magnetic and longstanding bartender, served as a backbone of the restaurant's familial culture. Over a period of a few months, however, Ken joined other bartenders in expressing frustration with management over their decision to add additional bartenders to every shift in order to improve service. Because bartenders pooled the money they earned from their own tips and the “tip out” money they received from servers, the managers' decision undercut their individual earnings. Given Ken's influence among staff, and even some managers, his dissatisfaction posed a risk to the restaurant's internal climate.

The tension came to a head one night when Ken was serving a regular guest: a bartender from a popular staff hangout down the street. It is customary within the industry to give a few freebies to valued guests, usually in hope that the act will be reciprocated through higher tips. At The Jones, this practice, known colloquially as “comping,” was officially accepted as a means of cultivating customer loyalty, although each case was ultimately up to managerial discretion. DJ, the manager on duty that night, had not approved any comping for Ken's guests, yet he noticed that “$70 worth” of food and drinks had been taken off the regular's check. He confronted Ken and was appalled by his response: “he basically said that he was comping all sorts of checks because he was angry at us for how we were scheduling, and he wanted to make more money!” Ken later clarified to other staff members that he said something along the lines of “the way you guys are staffing, if I don't take care of my regulars, I won't make enough money to live.” Nevertheless, in what another bartender described as “corporate deductive reasoning,” Ken's actions were interpreted as insubordination and theft, and he was promptly fired.

Shockwaves rippled through the restaurant. Over the next week, staff members made a highly visible display of collecting funds to support Ken's “livelihood.” When one staff member worried aloud that Ken might think the collection was “pity” money, another quickly rebuffed the idea, saying, “no, tell him we are mad, and we think this [incident] is stupid! This is our way of showing our support and standing up for him.”

In reconstructing the story for readers, the ethnographer accumulates multiple sedimentary facts that, individually, are not very material. A fact-checker may try to investigate the cash value of Ken's comped items, confirm the details of the exchange between Ken and DJ or seek to uncover both the formal records and common understandings of the comping policy at The Jones. Doing so, however, would be largely inconsequential to Mosseri's finding that people use money, as a cultural resource, to create boundaries and advocate for their own interests within workplace cultures that emphasize interdependence and community.

Through their public collection for Ken, for example, The Jones' staff drew upon the normative perception of money as depersonalized and fungible to reduce their ties with management to economic terms, countering the restaurant's family ideal and demonstrating that—as solely a source of income—employers, like workers, could also easily be replaced. Ken and DJ's altercation further shows how each deployed distinct social meanings of money to reframe the tumultuous intimate relations of conflicted workplace interests, with DJ viewing wages as a reward, earned through hard work, and Ken viewing wages as his livelihood and thus a right not to be denied. In the former view, “comping” is a practice that has the potential to align worker and customer in a way that threatens meritocracy and consequently workplace hierarchies, while in the latter, the practice serves the needs of all parties: workers through better tips, customers through freebies and the company through improved loyalty. Fact-checking the sedimentary facts of this case loses sight of the larger point that emerges from careful, contextual analysis, akin to debating the brush strokes in an Impressionist painting.

The last kind of fact that challenges the checker but is common to ethnography is what we might call a *motivational fact*. Motivational facts do not necessarily matter if they are correct, because what is important is that the informant believes they are; their inclusion builds the evidentiary base for richly textured multiple meanings generated by cognitive empathy. When Utrata (2015) talked to fathers in Russia for her book *Women Without Men*, for example, she noticed that they swore by a very low bar for what constituted an adequate family man. “As long as his children don't feel like orphans,” one informant told her (201); others insisted that all men are unfaithful (195). Men's low bar for fatherhood contributed to a gender crisis that has swollen the ranks of single mothers and reflected the marginalization of men in families, Utrata argues. “In the realm of family life, the negative cultural discourse on men creates a self-fulfilling prophecy,” she concluded (229). Whether or not all Russian men are unfaithful is immaterial; what matters is that Russian men believe it to be true. As Wedeen wrote, “When Scott (1985) analyzed how poor peasants and landlords recounted events, for example, he was less interested in whether their narratives were true than in how the disagreement worked to constitute a moral economy of village life” (Wedeen, 2010, p. 267).

Yet what matters with the motivational fact, the reformer might protest, is not the accuracy of the statement but whether the person said it, which could be verified by reviewing interview transcripts. This poses an enormous logistical problem, of course. If we are supposed to be worried about an ethnographer lying in their manuscript, where they include an abundance of data and information on methodological considerations, why should they stop there? What would prevent them from fabricating transcripts or field notes or creating “deep fake” recordings? More involved fact-checkers could also contact some sample of informants who—assuming they remember and will own up to it—could confirm that they said what the transcripts said they said (e.g., Desmond's, 2016). Yet these steps are once again not just prohibitive, but presume that the situation in which such statements arose—the relationship between ethnographer and informant, the relationships between participants, the context for their speaking—is immaterial.^4^

In actuality, the shaping power of researcher and context is considerable, and this is even more critical in the case of fieldnotes. These ethnographic artifacts are neither self-explanatory nor complete, far from flat documents easily transferred from one researcher to the next, but rather notes that come to life in relation to the researcher (Reyes, 2020). The valuable physiological knowledge produced through ethnographic immersion is rarely explicit within the ethnographers' fieldnotes. This tacit and embodied knowledge may not even be fully accessible to the researcher at the time but instead is made available later through self-reflective analysis and writing.^5^

Most important, the facts outlined above—those that pose such challenges for fact-checking—are exactly those that speak to ethnography's multi-vocality. The conflict, the irony, the wrenching emotion of the contradictory fact, the richly detailed puzzle pieces of the sedimentary fact, the motivational fact's claim to representing particular voices—these are foundational to ethnographic claims and to the value of qualitative work (Small and Calarco, 2022).

To be sure, fact-checkers by themselves do not eradicate these contradictions, and it is of course possible to produce multi-vocal accounts while also—we would argue despite—using fact-checking, Desmond's (2016) *Evicted* being a paradigmatic example. Nonetheless, the calls for fact-checking participate in an overall fetishization of individual “facts” as if they were static, immutable, singular and inalienable, ignoring their complexity, flexibility and polysemy, not to mention the connection between ethnographer and participants that helped to produce them. As we argue below, that complexity is part and parcel of resonance, and one of ethnography's core contributions.

Given the various ethnographic facts reviewed here, the use of fact-checkers is not very feasible, not very consequential, not very warranted or not very kind. Indeed, their use can seem more like a talisman adopted to reassure gatekeepers (mostly funders or editors) who do not understand or trust qualitative research methods. But by deferring to their suspicions rather than educating them out of them—increasing their qualitative literacy—adopting such talismans only delays the reckoning of the value of qualitative methods and their contributions, posing risks for the multi-vocal complexity that comprises ethnographies' core value and furthering rather than fighting the ethnographic marginalization reformers fear.

### Transparency's myth of neutrality

In recent years, practices used to protect research participants' privacy have come under fire. Most notably, Jerolmack and Murphy (2019) argued that “disclosure, not anonymization, should be the default convention within ethnography” (802). The authors warned that “masking” provides a false sense of security given an inability to fully guarantee confidentiality, threatens participants' voice and agency in cases where they prefer to be named, and may even undermine opportunities for knowledge production. More recently, the authors toned down their argument in response to challenges raised by other ethnographers (e.g., Stuart, 2016; Reyes's, 2018; Contreras', 2019; Seim, 2020), suggesting instead that researchers “anonymize as minimally as possible” (Murphy et al., 2021, p. 49). These arguments have gained traction, with some ethnographers appearing to provide a wholesale endorsement (e.g., Timmermans, 2019). Other ethnographers are more wary of naming practices but have nevertheless offered up concessions, e.g., Contreras' (2019) proposal for partial disclosure, Small's (2018) call for a “pragmatic approach to confidentiality” (197), and Reyes's (2018) case-by-case framework for decisions regarding transparency.

We contend that the terms of this debate are fundamentally uneven. “Masking” implies suspicious practice, while “transparency” ostensibly conveys objectivity, and we take issue with both. Ethnographers do not begin with the goal of obscuring information; their orienting framework—or baseline consideration—is to reduce participants' risk, and anonymization is one of the only and, while not foolproof, one of the most effective tools they have to do so. Moreover, transparency is far from a neutral broker of truth, and portraying it as such ignores or significantly downplays inequalities in privacy and the dangers of visibility for marginalized groups that have been chronicled by many (e.g., Lyon, 2003; Monahan, 2008). Given the unevenness in this debate, and the pressure it places on scholars to adopt reforms, we find it necessary to detail why naming practices are problematic.

Advocates of naming practices argue that ethnographers can never guarantee participants' confidentiality, especially in the Google era (Scheper-Hughes, 2016; Lubet, 2018; Jerolmack and Murphy, 2019). This risk of unintended disclosure is real, and it is something that ethnographers explicitly consider and take steps to minimize, as codified during the IRB approval process. The risk of disclosure should also be, and typically is, discussed with participants during the consent process to quell a false sense of security. We agree that none of these steps guarantee participants' protection from harm, but we argue that that does not make the overall effort unworthy. Declining to try to conceal identities because to do so has become too challenging in the information age punts the responsibility of protecting participants to participants themselves. Moreover, it denies participants the potential for plausible deniability. As Reyes's (2018, p. 212) writes, “it is one thing to guess at someone's identity and another to know for certain who those people are.” Plausible deniability provides even known participants with some insulation from the potential consequences stemming from findings disclosed.

Reformers also question the very premise that confidentiality is desirable for participants, noting cases where research participants may want to be named and may, in some situations, enjoy material benefits due to their heightened visibility (Duneier, 1999; Jerolmack, 2013; Broughton, 2015). Masking thus undermines such participants' agency and voice. This argument, however, oversimplifies agency and consent. For one, it overlooks the interconnectedness of participants, how one individual's request to be named forces the hand of other participants, some of whom may have more to lose. Moreover, unmasking does not allow participants to change their minds about being known.

Lareau's (2011) revisit to the families that participated in her influential study, *Unequal Childhoods*, provides an illustrative example of how participants' feelings about their association with a study may evolve over time. The families expressed displeasure with the book, but as Lareau notes, “accuracy was not the crux of the problem. The problem was how the families felt about the way they were portrayed” (326). For example, the mother featured in the chapter entitled, “Beating with a Belt, Fearing ‘the School': Little Billy Yanelli,” thought that the book made her family seem like child abusers. Originally, the Yanellis were excited about the study, anticipating that the book was “going to be like the book Oprah had”; little Billy “had been looking forward to showing people about the book but now he felt he couldn't show it to anyone” (323). These reactions suggest that this family might have sought for Lareau to use their real names if she had made that option available to them, a decision their later comments suggest they would have greatly regretted. The change in their views is a crucial point. Importantly, that change is possible not only prior to publication but also once the research is out in the world, taking on new life as the surrounding social context evolves. Masking makes it possible for informants to act in accordance with their revised views. They retain the ability to show other people the book, or not, and their confidentiality enables them to keep an arms-length distance to any public dialogue surrounding its findings. Disclosure, in contrast, would make informants' felt regrets more common and more acute, largely, we would add, in service to future researchers' potential convenience.

*Unequal Childhoods* offers rich ethnographic evidence for a set of powerful theoretical findings about how parenting contributes to class reproduction. Social science (and society, we would argue), is surely better off for this book having been written. Yet the costs to the families involved appear to be in some cases fairly high, particularly in their embarrassment and chagrin at what they look like in the book [a point noted by Jerolmack and Murphy (2019)]. As it stands, they are able to wrestle with these emotions on their own terms, out of the spotlight and without potentially significant or long-term consequences associated with public condemnation. Had their identity been known to readers, those costs would undoubtedly be higher and more prolonged.^6^ Neither researcher nor participant know how a book's portrayal will be received; that very uncertainty means it is difficult to control when transparency in research might turn into surveillance of the researched. Public recognition can incur real risks, particularly in an era of trolling and doxing and particularly for communities susceptible to social policing, such as women and people of color (Gosse et al., 2021). These risks are borne largely, but not solely, by participants, who are much more vulnerable than researchers, and for whom the individual benefits are often less. Their vulnerability, and the gift they offer in their engagement with our research, obligates us in the strongest terms to protect them.

Lastly, reformers argue that anonymization practices sacrifice opportunities for scholarly reanalysis, which Murphy et al. (2021, p. 4) define as the marshaling of “any and all available data to independently evaluate an ethnographer's interpretations and consider alternative explanations,” and which can take the form of ethnographic revisits, comparisons to large n data sets and other primary sources, or secondary analysis of field notes. Without the transparency provided *via* naming, they ask, how can scholars pull out sociologically relevant details, determine how to generalize or develop comparison studies? To take the case of revisits, this practice certainly offers intellectual value (Burawoy, 2003). Yet, we urge caution for reasons of both ethics and scholarship. In ethical terms, revisits might veer into exploitative if people feel compelled, because of their (or their predecessor's) involvement in past research, to participate in future studies seeking to build upon the original. Prolonged research increases the costs of participation, requiring continued attentiveness to the (already uneven) distribution of risk and benefit between researcher and researched. As a collective, we need to make sure that the places and people from which we draw knowledge are not being unnecessarily tapped over and over.

Regarding the value of revisits for scholarship, any revisit to a site involves a new historical moment, and often a new researcher, which means they do not generally provide opportunities to “double-check” the original ethnographers' empirical observations, especially given the hard-to-replicate path dependencies created through the qualitative research process^7^; indeed, we echo Pratt et al. (2020) in reminding scholars not to “conflate replication with trustworthiness” (1). Furthermore, unmasking may not do much to reveal that which was missed by the original observer and of interest to the new ethnographer. We argue parallel studies in a new location or among new subjects may prove equally valuable in searching for negative cases or exceptions, leading to further theory development when found or greater generalizability of the theory when not. For example, despite masking, Kanter's (1977) study of “Indsco” generated numerous subsequent studies, such as those by Williams (1995) and Wingfield (2009), which made valuable revisions to her original theory of tokenism at work. Indeed, we note that Wingfield's study introduced a racial analysis to productively modify the original theory, despite the limited information provided by Kanter on the race of Indsco workers and managers.

Ultimately, we argue, calls for reform seem to ignore the politics of transparency and fetishize ethnographic fieldnotes as “facts,” easily adopted for alternative use outside of the context in which they were produced. Moreover, this discussion fails to acknowledge how ethnographies already prioritize data elaboration *within* the text, a stark contrast with quantitative, hypothesis-testing research, where data reduction is pursued to reduce confounding noise and enable standardized comparisons. Indeed, we contend that unmasking impedes this more robust transparency within ethnographic work, as it treats identities as largely fixed, not shaped in tandem with their social environments, bearing implications for multivocality and the ability to honor the fluidity and complexity of human life and emotion. The pitiless glare of notoriety is not conducive to the nuance, flexibility, and ambivalence of ethnography, which we consider its greatest strengths. Unmasking exposes informants to demands for narrative and emotional coherence, and shames those who are forced to bear witness to their own compromises. Voices would be less likely to haunt an unmasked ethnography, and instead simply ring forth with positions people are not afraid to espouse under the gaze of others. Instead of unmasking, we encourage ethnographic readers to pay attention to “follow-up” (Small and Calarco, 2022): is there evidence that the researcher probed on statements and events that were confusing? This real-time practice honors the emergent aspects of ethnographic data that enrich the account.

Reforms stemming from the perceived credibility crisis have a number of different problems, but most critically, they reduce ethnography to its component parts—facts and names and typologies—and interrogate each piece for some inner truth. To give too much weight to these verifiable pieces, especially over and above the more holistic narrative presented by the ethnographer, would be akin to claiming that “a birth certificate is a birth, or a script is a performance, or a map is a journey,” an error shrewdly highlighted by British author Mantel (2020) in a Reith Lecture. In short, these reforms challenge the complexity that makes ethnography valuable, and of note for this paper, their capacity for resonance.

## Resonance and ethnography

If proposed reforms threaten what we think is most valuable about ethnography—its capacity for resonance—our discussion about the reforms would not be complete without outlining the concept of resonance, how ethnographies accomplish it, and how the reforms impede it. In what follows, we build upon recent scholarship in cultural sociology to elaborate and refine a “textured” model of resonance as an example of what ethnography does well. By outlining this concept of resonance, we seek not just to demonstrate how we evaluate better or worse ethnographies but also to illuminate the broader value of good ethnographic research for social scientists, and ultimately, for society.

The history of the concept of resonance has largely been situated within social movement scholarship (e.g., Snow and Benford, 1992) and in research focusing on the cultural reception of music or art (e.g., Binder, 1993), but has been plagued by the lack of a shared definition (McDonnell, 2014). Moreover, some of this earlier work seemed to conflate resonance with relevance: resonance reflected a connection between a cultural message or symbol and an audience with interests that were socially constituted beforehand (e.g, Schudson, 1989). For Schudson, for example, cultural objects obtained resonance in part from how their audience was able to put them to use, as informed by how these objects interacted with prior traditions. “In this view,” write Hallett et al. (2019, p. 548), “a social science idea would have ‘resonance' with the public to the extent that it fits their worldview, experiences, and expectations.”

More recent scholarship has tackled some of these limitations. In a series of publications, McDonnell (2014; 2016; McDonnell et al., 2017) offer what we might term a “pragmatic” model of resonance, through which they usefully add a needed dynamism to the model, resolve the tautological quality of earlier definitions, and suggest ways to measure it [see also Glaeser's (2011) discussion of “resonance in pursuit”]. The “pragmatism” of the model is one that locates the “point” of culture in helping people solve problems, broadly construed. A resonant object, they contend, “may crystallize a previously unarticulated experience, provide a novel way to approach a problem [that] actors routinely encounter, or actually problematize something previously taken for granted in a way that sheds new light on an old pragmatic problem” (2017, p. 4). When people have emotions they do not know what to do with, for example, a well-timed ethnography can help to “solve” that conundrum by offering clear reasons for those sentiments, for example (e.g., Bonikowski, 2017).

The benefits of the model are several. First, McDonnell et al. (2017) urge a particularly dynamic approach, arguing that resonance is not a fixed trait that cultural objects have or do not have, but rather it is an attribute-in-relation that emerges in a given cultural context and can later subside. Their model places a resonant cultural artifact not only *within* the specific relationships between author, object and consumer but also *at* a particular time and place. Second, they argue that resonance is about more than just an echo of what we know already, but rather a means of connecting what we know to what we do not. Cultural objects become resonant as they help audiences make sense of their experiences and interactions, and so they feel like an “aha” moment, “heightening emotions and enabling actors to transcend what was previously taken for granted” (McDonnell et al., 2017, p. 4). Finally, McDonnell (2014) adds some helpful means of operationalizing resonance—in the heightened state of emotions with which people greet or absorb the idea. These contributions have spawned a renewed interest in resonance, and scholars have found the approach fruitful, applying it to a range of studies such as how social science contributions become “public ideas” (Hallett et al., 2019), how organizations appeal to volunteers (Paxton et al., 2020); and how radical right politics mobilize collective resentment (Bonikowski, 2017). Interest in resonance spans multiple sociological subfields.

Understood in this way, it becomes clear that providing resonance is also at the heart of what ethnographers seek to do. Ethnographies reflect the social world, but through the analysis, reassemble it in a new way, making the strange feel familiar or the familiar feel strange. Hochschild's (2016) *Strangers in their Own Land* is a good example: it offered an illuminating metaphor for the seemingly irrational contempt of government among those with the greatest need for its help as akin to the everyday, shared frustration of waiting in a line that, for various reasons, fails to progress. Ethnographers use familiar chords to bring new sense-making tools to readers, and through both alignment *and* transcendence, they achieve resonance with readers. As the anthropologist, Messeri (2017) describes it, resonance is how “the knowing and sensing subject”—whether that be the ethnographer or the reader—“detects and amplifies connections between discrete, distant objects and worlds” (132). She explains that resonance “brings closer the conceptually distant worlds that culture tends to reify” (140) and “allows humans to know one another” (133). This result reflects the more dynamic understanding of resonance that McDonnell et al. (2017) suggest, in which the cultural object not just echoes but reconfigures or expands.

At the same time, however, there are limits to what the pragmatic model can explain about resonance in ethnographies. First, its insistence on the practical utility of resonant cultural ideas or objects shares a generative tension with the robust finding of the ambivalence or multi-vocality of compelling cultural objects (Reed, 2011). It is the very flexibility of meanings that allows certain ideas to speak to large audiences, since, as Schudson (1989, p. 159) noted long ago, “no cultural objects work with everyone, none of them affects even the people they do affect in the same way.” It is not that the pragmatist emphasis on problem-solving and this kind of flexibility inherent in resonance are contradictory exactly—we can imagine that rich, complex cultural ideas might allow their audiences to express their own ambivalence, which also “solves a problem” of sorts. In addition, the ability to re-apply a cultural idea to a new situation is part of the “interpretive flexibility” of particularly “public” ideas, according to Hallett et al. (2019); [see also Vaughan (2006) re: the ethnography as “boundary object”]; McDonnell et al. (2017) make room for this kind of periodic renewal with their dynamic approach to resonance-as-process.^8^ Yet viewing cultural objects as “solutions” suggests a certain fixity to their meaning-making, and rather less flexibility than more.

This points to a larger flaw regarding the pragmatic approach: problem-solving, even when broadly construed, takes as its focus “problems,” and intimates that they are overcome; it creates a dyad between problem and solution. Yet, the social world is much more multidimensional and complex—we might sit with problems or worry them like a bone; dilemmas can create motive or structure; we may be anxious or unmoored by them or even enjoy them. Of course, pragmatists might counter this point with the notion that solutions also are plural and not one-dimensional. Nonetheless we maintain that the issue here is not how complex the solutions are, it is that the very definition of problem-solving seems to set the world into two binary categories and thus threatens the heterogeneity of the study's findings.

Second, we would argue that the pragmatic model offers a fairly limited role for emotions. The model seems to look to emotions as a stimulus for resonance, but emotions are also a medium for resonance, which we consider a crucial distinction, albeit challenging to parse operationally. The former notion of emotions-as-stimulus prompts resonance, but resonance remains largely cognitive. The latter emotions-as-medium conceptualization argues that resonance is an emotional process, at least in part. In addition, while the pragmatic model usefully invokes an emotional dimension to resonance, its authors seem to insist on only a positive view. In his earlier article, McDonnell (2014) asserts “a strong connection to positive affect,” arguing that the content of the “aha” moment “matters tremendously,” and that resonance means not fear, horror or shock, but ebullience (262). Later, McDonnell et al. (2017) distinguish between salience (“when an object or idea becomes a social problem”) and resonance (“when objects and ideas *solve* practical problems”) (9). Yet not all examples of resonance invoke solely positive emotion. If we agree that Bonikowski (2017) is analyzing the resonance of radical right ideas, for example, the collective resentment that they harness is very related to fear, horror *and* perhaps a certain ebullience (see also Lamont et al., 2017).

These differences, while partial, are important; they also center on the unique potential for resonance in ethnography. One of the primary strengths of ethnography is in its capacity to convey and elicit emotional ambivalence, contradiction, and the multiple meanings of many voices. In this way, resonance is both a tool for researchers in their work and a product of their work (Messeri, 2017). To adequately center these contributions, we develop what we term a “textured” model of resonance.

In this model, resonance is, as the literary historian Stephen Greenblatt (2018 [1990]) argues, “the power of the displayed object to reach out beyond its formal boundaries to a larger world, to evoke in the viewer the complex, dynamic cultural forces from which it has emerged and for which it may be taken by a viewer to stand.” Note that this definition does not differentiate between personal resonance and what we might consider collective resonance, or appeal to a large audience; we share this agnosticism about resonance's scale. More important, resonant objects are multidimensional, containing diverse voices and complex histories, often excavating difficult emotions while also potentially moving audiences toward a longing to overcome what society is, has been, or could become.

As an example, Greenblatt describes an exhibit of Judaica from communities across Moravia and Bohemia, housed in the Prague State Jewish museum, which was distributed across several area synagogues, including the “Old-New synagogue” from the 13^th^ century. Contrasting resonance with wonder, which he defined as “the power of the displayed object to stop the viewer in his or her tracks, to convey an arresting sense of uniqueness, to evoke an exalted attention” (Greenblatt, 2018 [1990], p. 265), Greenblatt notes that the objects themselves are rather ordinary and not particularly arresting aesthetically. Their resonance, he argues, “depends not upon visual stimulation but upon a felt intensity of names, and behind the names, as the very term resonance suggests, of voices: the voices of those who chanted, studied, muttered their prayers, wept, and then were forever silenced” (268). The voices belong to Jews murdered in World War II, Greenblatt writes, but also to those massacred while seeking refuge in the Old-New synagogue in 1389.

Yet the complexity does not stop there. The museum's “ultimate source of resonance,” Greenblatt (268) argues, is that it was the Nazis who amassed the bulk of the collection. “Most of the objects are located in the museum—were displaced, preserved, and transformed categorically into works of art—because the Nazis stored the articles they confiscated in the Prague synagogues that they chose to preserve for this very purpose.” Abused and malnourished curators were tasked with organizing and displaying these objects for SS officers' private viewing, until they themselves were rounded up and sent to the camps to die. After the war, the Jewish community donated the objects to the state for their preservation, Greenblatt reports (269), creating the “resonant, impure “memorial complex” they are—a cultural machine that generates an uncontrollable oscillation between homage and desecration, longing and hopelessness, the voices of the dead and silence.” Resonance is not about consensus nor does it resolve; its captivation relies, in part, on the tension—the constant oscillation between competing ideas and emotions—that it evokes.

Thus the conventional definition of resonance that prevailed for decades—as that which confirms what audiences think already—fails to capture the complexity that makes resonant ethnographies so powerful. The pragmatic model improves upon this original idea with a hybrid vision of new and old—the aha! epiphany. But even so, as noted above, scholars intimate that resonance comes with a sense of new clarity. Ultimately, the pragmatist priority—culture must solve problems for its users—seems a bit awry here, if not actually wrong. Resonant ethnographies do more than explain, they raise curiosity, leading us to ask particular questions, look for particular clues, notice particular details. When we move beyond a problem-solution binary, we find ourselves able to hear a bit more from a scene: the sometimes many contradictory voices, the irony, the complexity, the multiple layers, aspects which deepen and enrich our experience and understanding.

These aspects also happen to be exactly what ethnography brings to the social science table. The textured model of resonance argues that sometimes resonance is not about the reach for clarity, suggesting instead that multiple layers of meaning—not all of which agree, or point in the same direction, or tell us to feel the same thing—contribute to resonance. To achieve resonance, ethnographies unearth contradiction, irony, poignance, or paradox (Vaughan, 2004); they exhibit multi-vocality. As Greenblatt writes: “the key [to resonance] is the intimation of a larger community of voices and skills,” what he calls (269) “an imagined ethnographic thickness.” Second, the emotions that resonance invokes, indeed relies on, are far from only positive, but instead a complex welter. Often the voices that lead to resonance are powerful not because they resolve a persistent concern, but because they haunt a given cultural object, akin to what O'Brien (2009) called “an inescapable scratchiness.” It is that haunting that makes for the persistent thrum, the reverberations that create resonance.

As an example, consider *Crook County: Racism and Injustice in America's Largest Criminal Court*, Van Cleve's (2016) ethnographic account of the Cook County criminal court.^9^ In accordance with the pragmatic model, *Crook County* became resonant within a particular cultural context. The book was published in 2016, at a time when white Americans were faced with a puzzle: evidence of enduring racism in police shootings and right-wing resurgence despite a colorblind ideology that peaked in the early years of the Obama presidency. In this perplexing moment, Van Cleve connected what was already known to relatively new insights: racism persists without racists; racism is not a pathology within institutions, it often serves a function for those institutions; the criminal justice system does not simply produce racist outcomes, it operates through racist processes. The narrative in *Crook County* offers some explanation of the persistence of American racism to a growing audience interested in understanding it.

Yet, the book's value involves more than explanation. The power of the narrative is driven by its complexity and multidimensionality. Van Cleve juxtaposes maddening and heart-wrenching stories of “racial degradation ceremonies” that take place within the court system with a patient immersion into the professional culture that fosters them. The reader bears witness to court professionals mocking, ridiculing, and verbally abusing defendants, especially poor people of color, many of whom entered the criminal justice system *via* false allegations or minor infractions. The narrative condemns the professionals' behavior and makes visible the intense suffering it produces, but simultaneously salvages a piece of their humanity. Van Cleve describes how professionals' devotion to justice is subverted within a cultural logic that renders the targets of their abuse as morally worthless “mopes,” distracting them from the more rewarding pursuit of prosecuting violent “monsters.” We learn how professionals rationalize their behavior within the broader system of justice and how most view themselves as neutral participants—if not allies—in the fight for racial equality.

*Crook County* resonates, not solely because it solves a problem, but because it excavates the complexity, the messiness, the irreducibility of life. The social world entails conflict, compromise, and an enduring lack of resolution, and ethnographies that resonate are like a prism, parsing the sunlight to reveal the multitude of color hidden within. Contradictions are not beside the point, they are the point.

## The power of reflexivity

We have argued that recent ethnographic reforms demand narrative coherence, impede flexibility and polysemy, and fetishize “facts” and “fieldnotes” as if they were static and immutable. These reforms confuse transparency with authenticity, constructing ethnography as a window instead of a prism, pursuing verification over interaction and achieving clarity at the cost of complexity. In sum, they sacrifice ethnography's resonance. However, there are already time-honored practices, honed through productive dialogue among practitioners, that address issues of credibility and trust in ethnography without trading off its central contributions. While reformers dangle such a costly path to legitimacy for ethnographers, we contend that if readers do not trust the ethnography by the time they finish reading, fact-checking or exposing names and places will not fix that problem. Instead, by that point and for those readers, the ethnographer has already failed in their task. Trust in ethnography is built incrementally, through practices of reflexivity.^10^

The three most important approaches to reflexive practice, as we consider them, are *pursuing radical self-consciousness, interrogating consensus* and *exploring inconvenient data*. There are other reflexive practices that are worthwhile, such as checking back with informants or listing anonymized participants and their relevant characteristics in the text; we view the three approaches as broader and worth discussing because many reflexive practices are encompassed within them. As they have been the subject of extensive scholarly conversation and in many cases reflect longstanding practice, our discussion here is necessarily abbreviated. Our point, however, is twofold: that ethnographers build trust bit by bit rather than simply rely on readers' faith, and that these practices do not generally risk other dimensions of value, such as resonance.

Radical self-consciousness, or the ongoing consideration of how one's identities, relationships, expressions and resources shape the research process, is replete in ethnographic texts.^11^ This practice focuses on the complexity, contradictions and sometimes changing perspectives that ethnographers can inhabit. Because ethnographers are themselves the instrument of data collection and analysis, they more continually confront the opportunities both blocked and made possible through their social location, and they are attuned to how their subjectivities and theoretical commitments shape the phenomena they notice and the insights they glean (e.g., Duck, 2015; Reyes, 2020). As established practice, ethnographers “constantly ask ourselves about our research design, our relationship with our research participants, the labels we give them, and the way we write about them” (Rios, 2015, p. 260). This practice is not extraneous to ethnography's conduct, “extras” that are somehow icing to the ethnographic cake; instead they are the cake, ethnography's widely shared norms, albeit imperfectly followed.

Interrogating consensus is when ethnographers check common phrases and practices for assumptions that erase other viewpoints, often centering the perspective of their site participants. A recent example of this strategy in action is Altomonte's (2020) research within post-acute care units that serve elderly patients recovering from hospital stays of three or more days. Altomonte finds that care staff are morally committed to the goal of “independent aging,” defined broadly as patients' return to autonomous life within their own homes. However, care staff toggle between different meanings of independence as they negotiate specific patient orientations within the competing mandates of safe and fast patient discharge that define their organizations. When trying to ensure safe discharge for patients perceived as being too hasty to return to their previous routines, for example, staff emphasize how *independence entails acknowledging one's limitations* and need for specific accommodations (e.g., walkers, sliding shower seats, at-home caregiver). In contrast, staff emphasize *independence as taking personal responsibility and achieving self-reliance* to prompt timely discharge when working with slowly progressing patients. By grounding the analysis in the lives of her participants—as opposed to existing social categories—Altomonte uncovers a complex and nuanced story that demonstrates how the ambiguity of moral concepts enables care staff to invoke seemingly contradictory logics at different points in time. Ethnographers interrogate abstract concepts, keenly aware of how universal language can erase participants' lived experience and agency, and they display skepticism toward systems of classification, known to be a source of symbolic violence (Bourdieu and Wacquant, 1992). In the field, they bring the taken-for-granted under scrutiny, questioning the “obvious” meanings of familiar vocabularies and practices (see Vaughan, 1996 for a paradigmatic example). This interrogative, skeptical curiosity is part of the ethnographer's arsenal in the bid for credibility.

A third approach to reflexive practice is the constant exploration of what Duneier (2011) calls “inconvenient” data. Inconvenient data are made up of examples—those negative or exceptional cases—that throw a wrench in the theories or arguments that an ethnographer might deploy. These anomalies are useful in that they can expose blind spots in the ethnographer's thinking, and they may prompt a productive reconfiguration of the analysis; they also add—rather than necessarily solve—contradictions or deviations in patterns that can enhance resonance.

The search for inconvenient data sometimes starts with research design. In *Pricing Beauty*, for example, Mears (2011) analyzes fashion modeling as a “deviant case” in which women outearn men. Traditionally, men working in feminized fields experience a “glass escalator” effect (Williams, 1992), in which they quickly move up within organizational ranks. In the case of fashion modeling, however, precarious, short and non-linear careers disrupt this process (Mears and Connell, 2016).^12^ The anomaly of the inverted wage gap also sheds light on how the objectification of women's bodies is culturally celebrated, while men's sexualized bodies are devalued. Women earn more in fashion modeling, Mears shows, but at the cost of reproducing pernicious cultural beliefs about gendered bodies. The pursuit of inconvenient data also occurs during data analysis (e.g., Thorne, 1993; Khan, 2011), making conclusions at once more refined and more nuanced as a result of the consideration of exceptions within the data. Like pursuing radical self-consciousness and interrogating consensus, exploring inconvenient data introduces multi-vocality and complexity to ethnography.

These widely practiced strategies of reflexivity bolster the credibility of ethnographic research and do so not by reducing but by maintaining complexity. Ethnographers use these and other approaches as an opportunity to explore and bring attention to multiple, coexisting realities, many of which are concealed by the processes of standardization and generalization common within other research methods (Collins, 1990; Harding, 1992). As Bourdieu and Wacquant (1992, p. 236) argue, “a scientific practice that fails to question itself does not, properly speaking, know what it does…it records itself without recognizing itself”. The upshot of this reflexive undertaking, however, is not to superimpose the standards of other methods onto ethnography; rather, we urge practitioners to pursue the kind of reflexivity that does not harm its capacity for resonance.

## Conclusion

Concern over ethnographic methods is burgeoning, propelled not only by controversies bedeviling recent examples of the trade, but by the credibility crisis roiling psychology and calls for greater transparency, replicability and access to quantitative data from within academia as well as the lay public. Reformers express worry about the marginal status of ethnography in a positivist discipline. Yet in seeking out a reluctant anointing from suspicious others, many of whom already view ethnography as not-quite-social-science, these reforms increase the vulnerability of ethnographic participants, and further, threaten to undermine what we consider what ethnography does well.

Instead, we join Small and Calarco (2022) in calling on practitioners to articulate and embrace ethnographic best practices, strengthening and developing them from within an interpretivist perspective. Ethnographic research enables rich accounts of social worlds and the perspectives of their inhabitants, helping to disentomb social life and reveal its dynamism. Crucially, ethnographic best practice includes striving for resonance by offering up concepts and practices that invoke multi-vocal, flexible meanings with interpretive depth and complexity, developing insights with “chimes that people feel down to their feet” (Lepselter, 2012, p. 101). These strengths are at stake in proposals that strive to nail down “facts” or name informants.

Ultimately, we also question whether the reforms would do much to shore up the legitimacy of ethnography among its critics. At best, it seems ethnographers would be settling for ill-fitting, yet universal standards of evaluation, akin to judging all movies—not just comedies—on whether or not they are funny. At worst, fact-checking and naming practices may provide critics with resources to further scrutinize ethnographic work that they do not like. Rather than depending on outside experts to establish veracity or expertise after the research is completed, however, ethnographers carefully bid for the confidence of their readers by adhering to common strategies of research conduct from beginning to end. Their efforts will not convince every reader. Yet these strategies do not depend on ex post facto stamps of verification from some external source, but instead work to establish trust in layer after incremental layer, through practices of reflexivity.

We are struck, in this debate about research credibility, by the tacit focus on individual researchers, as if they alone are the problem-maker or savant. We view this approach as asociological, ignoring how perceptions of trustworthiness are shaped by relations of power and inequality (i.e., Cook, 2005; Gambetta and Hamill, 2005; Ridgeway, 2009) and potentially leading some researchers—namely, early career researchers and those underrepresented in the most secure positions of academia—to be more vulnerable to scrutiny than others. Suggested ethnographic reforms, such as attempts at replication or calls for fact-checking, may as a result disproportionately target less powerful or historically marginalized scholars. Due to their social position, some scholars may therefore feel greater pressure than others to adopt practices like fact-checking, unmasking and sharing fieldnotes that are costly in terms of money, time and even physical safety (e.g., Reyes's, 2018; Contreras', 2019).

Alternatively, we encourage a more collective conceptualization of the problem, as well as its solutions. In an era in which many social science disciplines are slouching toward a methodological uniformity, shunning or defunding non-statistical approaches, one of sociology's strengths is its unique commitment to methodological omnivorousness. We need to match that commitment with institutional changes in training. Hancock et al. (2018) report that only 20% of top-20 sociology departments require a qualitative methods course of its graduate students—of which ethnography might occupy 1–3 weeks—while all of them require a quantitative course. Improved qualitative training would better enable fruitful methodological debate and strengthen peer review processes.

Anticipating that some may interpret our discussion of resonance as an appeal to popularity, we point out instead that the textured model of resonance is not fueled by commonality or even *a priori* alignment; rather, the heart of resonance within the textured model is the establishment of a meaningful connection. This connection, or relationship, can just as easily be derived from difference and disruption as from consensus. In this way, resonant texts respond to Abbott's (2007) call, within his “lyrical sociology” manifesto, for texts that confront us, as readers (and as authors), with “the radical chasm between our own here and now that of its subjects.” In revealing this difference, argues Abbott's (2007), “the chasm itself is crossed by our moral recognition of the common humanity we share with those we read about” (95). Resonant texts do not bow to popular morality, but by unearthing conflict and contradiction, they can—and frequently do—spark a moral consciousness that can bring us together.

Others may argue that resonance privileges style and form over substance and veracity—that captivation is in tension with truth. Yet, ethnographers' respect for inconvenient data, as highlighted above, belies this notion. Like some of the world's most celebrated artists, the best ethnographers, we contend, view dissonance as a resource, adding complexity, depth and drama to the work, not as an impediment to its beauty or even its coherence.

This critique also echoes longstanding (gendered) debates about emotion and rationality, subtly implying that the emotional dimension of textured resonance—the haunting, the “thrum” of feeling—can shape how an audience evaluates an ethnography, inciting passion and overcoming uncertainty. We have neither the space nor desire to rehash these debates here; suffice it to say, one is no easier misguided by a text that moves them than by one that relies on clinical but faulty or homogenous evidence. Indeed, we encourage greater attention within the discipline to what elements of truth may be lost with the latter.

A final potential limitation or downside of textured resonance as a feature of ethnography is that the fluidity and multivocality of resonant ethnographies may make them vulnerable to cooptation by politically motivated actors. Resonant ethnographies are complex and nuanced, stitched together to produce a *sui generis* patchwork. Purposefully fragmented findings from ethnographies risk misrepresentation when depicted in isolation within citations, media coverage or everyday talk. We would argue, however, that the fetishization of facts within proposed reforms are likely to promote—not prevent—the fragmentation and cooptation of ethnographic findings.

While we have focused on staving off reforms that we believe address a credibility crisis that ethnography does not have to own, we want to conclude by making a claim for the sheer value of trust as a practice. The merit of preserving trust in academic work seems particularly relevant in an era when many Americans feel that their confidence in social institutions has been betrayed. There are worthy practices and activities that enrich our world but that fundamentally at their core depend on a modicum of trust. There is a kind of leap of faith that is necessary to bring this sort of work into the world, and that faith is worth defending for the work and insight it makes possible. This is not to say that ethnographers should not work hard to demonstrate that their conclusions are sound—they should, and as we have shown, they do. At some point, however, the risk of deception is turtles all the way down, with manipulated records supporting manipulated texts. Transparency does not eliminate deception, and in fact, it can legitimate it by giving a false perception of disclosure. Ultimately, ethnography's distinctive contributions, as well as the substrate of trust on which all academic work ultimately depends, show us the importance of trust and trustworthiness for the sociological enterprise.

## Data availability statement

The data analyzed in this study is subject to the following licenses/restrictions: interview transcripts and fieldnotes are not publicly available. Requests to access these datasets should be directed to sarah.mosseri@sydney.edu.au.

## Ethics statement

The studies involving human participants were reviewed and approved by University of Virginia's Institutional Review Board for the Social and Behavioral Sciences (IRB-SBS). The patients/participants provided their written informed consent to participate in this study. Written informed consent was obtained from the individual(s) for the publication of any potentially identifiable images or data included in this article.

## Author contributions

AP developed the first iteration of the conceptual framework and the first draft of the paper. SM substantially revised the draft for critically important content. All authors have continued to revise and refine the draft.

## Footnotes

^1^This task becomes urgent when university press boards and journal referees start to call for the widespread use of fact-checking, heedless of the problems such tactics incur; some editors have noted privately to us, and with perturbation, that they are starting to hear more of these calls.

^2^While quantitative researchers have long incorporated inductive and interpretative elements into their practice, this work has often been obscured by writing formats and publication structures that ascribe to canonical social science's narrow ideal of objectivity.

^3^Some might argue that informants face that pain later, when they read about the contradictory fact in the published work, but as we argue below, they are able to do so privately and on their own terms.

^4^It is also worth noting that fact-checkers and the tools they use for fact-checking also exist within specific relationships and contexts. Relevant for this discussion is Tripodi's (2022) research into how conservative evangelicals do a lot of their “own research” to confirm discursive claims that exist in the public realm and the ways that seemingly objective search engines, like Google, echo back what these fact-checkers already believe based on the particular keywords used in their search.

^5^This point about analysis also raises the question: when does data sharing in the name of transparency begin to impede upon researchers' intellectual property rights, increasingly a matter of concern in a post-COVID era when faculty are being asked to generate materials that universities then commandeer?

^6^To their credit, Jerolmack and Murphy discuss the Lareau case in their 2019 article urging ethnographers to reconsider masking as default practice. Citing ethical concerns, they argue that the fact that the families were unhappy suggests that masking did not protect them emotionally. Yet ethical pursuits are not limited to the eradication of harm but also to its reduction. As the sex columnist Dan Savage once said (speaking about monogamy), ethical practices are more like sobriety than they are like virginity – something to keep striving for, rather than something that once lost is gone forever.

^7^See Burawoy, 2003 for a useful discussion of two such attempts at refutation and how they were met with defenses based on the subjectivity of data: Freeman's (1983) revisit of Mead (1929) and Boelen's (1992) critique of Whyte (1943).

^8^The Salem witch trials, for example, achieved a certain resonance in their day. According to Reed (2015, p. 87), they managed to corral public opinion through a process he dubbed “resignification,” which “binds consensus by suppressing alternate interpretations of the course of events.”

^9^Crook County was also the recipient of many accolades: among these, the American Sociological Association's 2019 Distinguished Scholarly Book Award, the recipient of the Society for the Study of Social Problems' (SSSP) Eduardo Bonilla-Silva Outstanding Book Award and a finalist for the SSSP's C. Wright Mills Book Award.

^10^While the new urgency to reanalysis usefully highlights the tensions of authorship (Murphy et al., 2021). Ultimately, we think, an ethnography represents one person's take, and these existing standards of reflexivity allow readers to see the author's positionality. We do not need a reanalysis of their ethnographic site to evaluate that.

^11^This concept is loosely akin to what Small and Calarco (2022) call “self-awareness.” As the authors note in their book, the concept of reflexivity varies substantially across the discipline and the social sciences more broadly. Our goal in providing this three-pronged overview is to add detail to discussions of reflexivity that might help to alleviate confusion and conflation within a complex and nuanced domain of academic writing.

^12^The authors also note similar dynamics among porn workers and strippers.
